# Challenges and diagnosis in therapy of secondary amenorrhoea in caesarean section patient with postpartum haemorrhage B-lynch sutures: a case report

**DOI:** 10.1097/MS9.0000000000001603

**Published:** 2023-12-15

**Authors:** Achmad Kemal Harzif, Putri Nurbaeti, Azizah Fitriayu Andyra, Budi Wiweko

**Affiliations:** Department of Obstetrics and Gynaecology, Faculty of Medicine University of Indonesia, Dr. Cipto Mangunkusumo Hospital, Jakarta, Indonesia

**Keywords:** amenorrhoea, asherman’s syndrome, B-Lynch, case report

## Abstract

**Introduction and importance:**

International Federation of Gynecology and Obstetrics (FIGO) and WHO report the incidence of postpartum haemorrhage (PPH) reaches 1–10% and contributes to an increase in postpartum maternal mortality with uterine atony as the most common cause. B-Lynch method is a suturing technique to overcome PPH. Although this method has proven useful as an emergency life-saving measure, the post-procedure complications are still able to occur.

**Presentation of case:**

The patient was not menstruating for 14 months after giving birth through caesarean section with B-Lynch due to PPH. Before pregnancy, she had regular menstruation cycle and normal menstrual duration. Her general and gynaecological status were normal. Ultrasound showed the impression of uterine hypoplasia and endometrium that were difficult to assess while both ovaries were normal. Diagnostic hysteroscopy showed a severe degree of Asherman’s syndrome. The results of FSH, LH and estradiol were normal.

**Discussion:**

B-lynch suture is performed as a method to stop PPH in uterine atony. Secondary amenorrhoea occurs as a complication of B-lynch. Compression action of B-lynch can cause progressive myometrium necrosis resulting in synechiae and blockade of uterine blood flow. This will interfere with the development of the uterus. Intrauterine adhesions and amenorrhoea with normal levels of FSH, LH, and estradiol support the diagnosis of Asherman’s syndrome.

**Conclusion:**

This case shows that the B-Lynch procedure, which is the worldwide recommended method for treating postpartum haemorrhage due to its high success rate, can cause complications of Asherman’s syndrome and cause secondary amenorrhoea.

## Background

Highlights

- Postpartum haemorrhage (PPH) is a complication in obstetric emergencies with an incidence which is namely around 1–10%. Its incidence continues to increase and becomes a major cause of maternal mortality.
- Mrs. F, 34 years old, came to the Obstetrics and Gynecology Polyclinic, Dr. Cipto Mangunkusumo Jakarta with complaints of not menstruating for 14 months.
- General and gynaecological status are within normal limits. Ultrasound showed the impression of uterine hypoplasia and endometrium were difficult to assess while both ovaries were normal. Diagnostic hysteroscopy showed a severe degree of Asherman’s syndrome. The results of FSH, LH and estradiol were normal.
- Compression action of B-lynch can cause progressive myometrium necrosis resulting in synechiae and blockade of uterine blood flow. This will interfere with the development of the uterus. Intrauterine adhesions and amenorrhoea with normal levels of FSH, LH, and estradiol support the diagnosis of Asherman’s syndrome.
- B-Lynch method is a suturing technique to overcome PPH and although this method has proven useful as an emergency life-saving measure, the post-procedure complications including Asherman’s syndrome are still able to occur.

Postpartum haemorrhage (PPH) is a complication in obstetric emergencies with an incidence which is namely around 1–10%. Its incidence continues to increase and becomes a major cause of maternal mortality. In 2017, the American College of Obstetricians and Gynecologists (ACO) changed the definition of postpartum haemorrhage to a condition of bleeding greater than or equal to 1000 ml, or bleeding accompanied by signs or symptoms of hypovolemia that occurs within 24 h after delivery. The most common cause of postpartum haemorrhage is 4T (four T’s) consisting of tone disorders (uterine atony), trauma (genital tract trauma), tissue (placental retention), and thrombin (coagulopathy). Uterine atony holds the highest percentage up to 80% in cases of postpartum haemorrhage. Uterine atony constitutes a condition characterized by inadequate contraction of the myometrium cells of the corpus uteri against endogenous oxytocin released during labour^1,2,3,4,6^.

If uterine contractions do not improve with non-operative methods, then operative management is carried out. Some of the surgical options that can be performed are the B-lynch suture procedure, uterine artery embolization, uterine artery and ovarian artery ligation, or hysterectomy. The B-lynch method is the most recommended technique in worldwide with a success rate of 91.7% in controlling bleeding. Although B-lynch suturing has proven to be very useful in treating postpartum haemorrhage, this method can cause various complications as well, for instance the development of synechiae in the uterus, tissue necrosis to abscesses. Approximately 25% of post-B-lynch patients in PPH develop complications within the first five years. However, this article shows the rare phenomenon of early complication due to B-Lynch procedure (14 months after procedure). Other complications that can occur due to compression suturing are uterine cavity occlusion, infection, pyometra, and infertility. Intrauterine adhesions accompanied by symptoms of hypomenorrhea or infertility are known as Asherman’s syndrome. Asherman’s syndrome is reported to be one of the causes of secondary amenorrhoea after the B-Lynch procedure^7–9^. This case report has been reported in line with the Surgical CAse REport (SCARE) 2023 criteria.

## Case

Patient came to the Obstetrics and Gynecology Polyclinic with complaints of not menstruating for 14 months on 15 November 2022. These complaints were felt after the birth of her first child. The patient gave birth to her first child on 1 June 2021, with a foetal weight of 3300 g, gestational age of 40–41 weeks, one nuchal cord, lack of amniotic fluid, and intrauterine foetal distress. The patient underwent labour induction, but was unsuccessful, thus a caesarean section was performed for indications of failed induction. During the intraoperative period, PPH was reported due to poor uterine contractions. Finally, the uterus was tied to stop the bleeding. Her menarche was at 14 years old with regular cycle of 28 days, duration of 5 days with 3–4 pad changes, and no pain during menstruation. The patient married once since 2020 until now. Additionally, there was no history of contraceptive use.

Her general condition is good; consciousness composmentis, blood pressure 120/67 mmHg, heart rate 77 beats/min, and respiratory rate 20 beats/min. General status was within normal limits. The gynaecological status showed that the urethral vulva was calm, without active bleeding.

**Figure 1:**
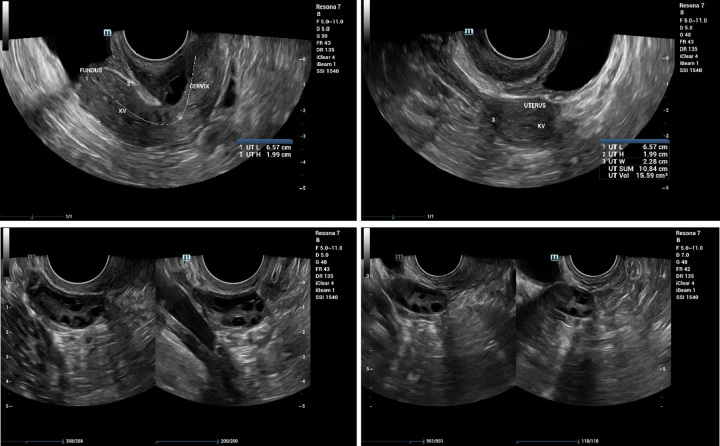
Transvaginal ultrasound.

The results of the transvaginal ultrasound examination (Fig. 1) showed a normal shape uterus with a hyper-anteflexed direction; uterine size was 65×19 × 22 mm with volume of 15.59 cm^3^; the homogeneous myometrium, difficult to assess the endometrium, slightly exposed uterine cavity, irregular surface, no intracavitary mass visible; endocervix and portio within normal limits; right ovary measuring 30×13 × 24 mm with a volume of 5.31 cm^3^, antral follicles visible, no dominant follicles visible, no masses visible; left ovary measuring 29×27 × 23 mm with volume of 9.59 cm^3^, no antral follicles visible, dominant follicle visible with size of 18×16 mm, no masses visible; both kidneys were within normal limits, no dilation of the pelvio-calyx system was found

Overall, there was an impression of a hypoplastic uterus, the endometrium was difficult to assess (adhesions were suspected), and both ovaries were normal. Furthermore, the patient was planned for hysteroscopy.

**Figure 2:**
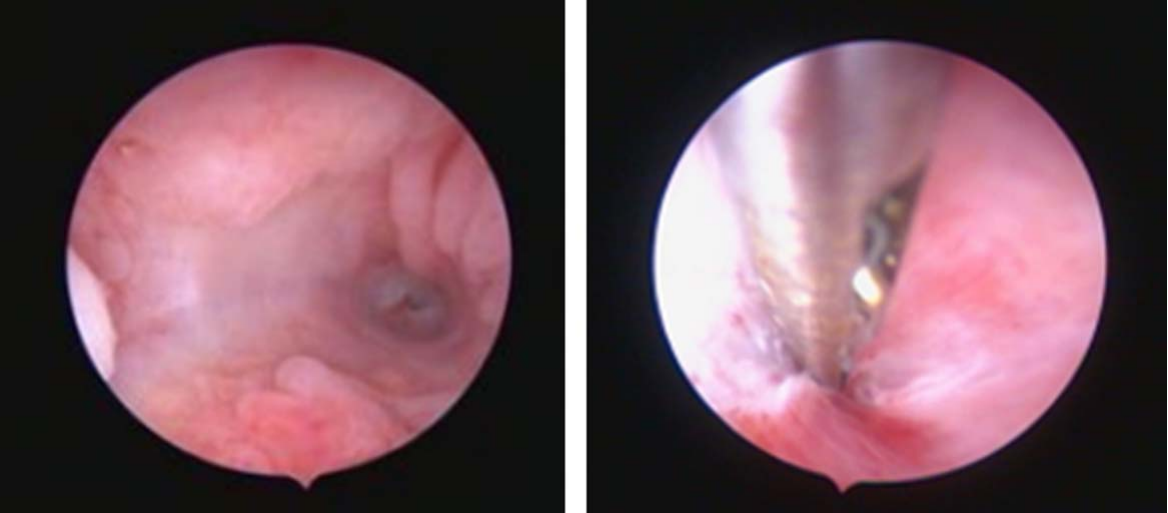
Diagnostic hysteroscopy.

As we see below (Fig. 2), the diagnostic hysteroscopy results indicated the walls of the vagina, cervix, and cervical canal were within normal limits; synechia was seen on OUI, probing was carried out using a fibrotic tissue impression grasper; a transabdominal ultrasound procedure with a scope view only reached the internal uterine ostium (isthmus), dilatation and dissection with scissors were attempted, but the patient was in pain so the procedure was stopped; left and right tubal ostium could not be seen. Diagnostic hysteroscopy results of severe Asherman’s syndrome

**Figure 3:**
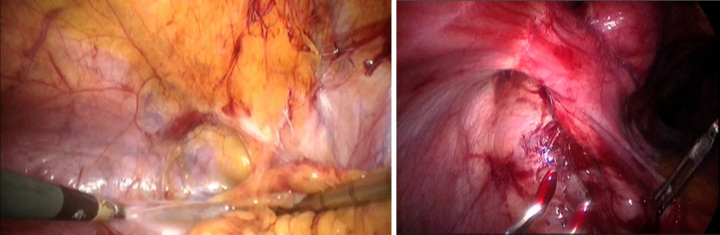
Laparoscopy of Intraperitoneal adhesion.

**Figure 4:**
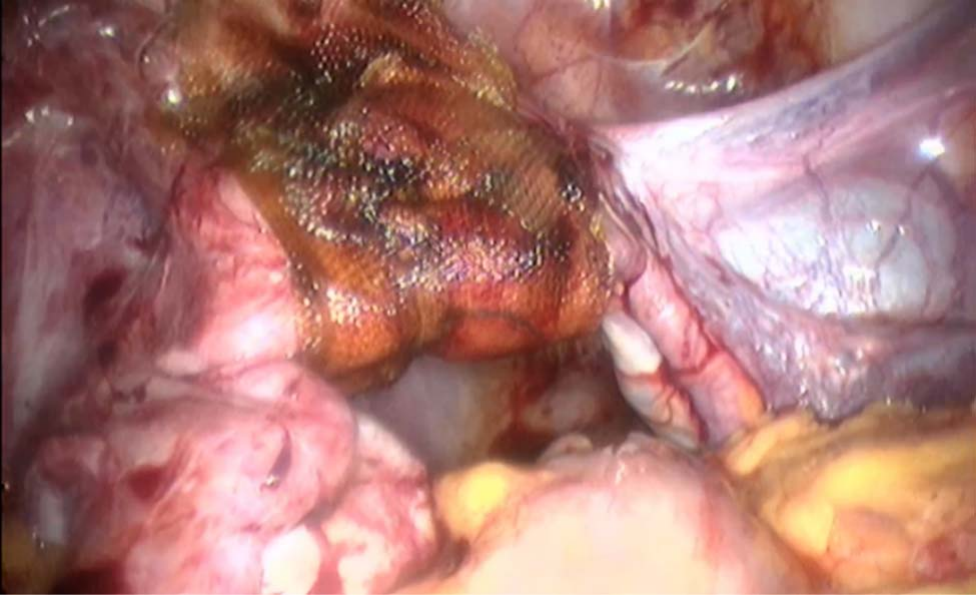
Post-adhesiolysis of uterus.

Patients were examined for FSH, LH, and estradiol. The three hormone tests were within normal limits, consisting of LH 3.0 mIU/ml, FSH 2.2 mIU/ml, and estradiol 143.2 pg/ml. Other laboratory tests are within normal limits as well. Based on the anamnesis, the results of physical and supporting examinations, the patient was diagnosed with severe secondary amenorrhoea which was Asherman syndrome and then planned for operative hysteroscopy as shown in Figs. 3 and 4.

After the diagnosis of Asherman’s Syndrome was established, the patient underwent surgical therapy with the patient’s consent on June 2023. The patient underwent hysteroscopic adhesion resection and laparoscopic adhesiolysis. The operation went smoothly without any complications. Follow-up postoperative were good. Patient was advised to have an IUD contraception, but physical examination showed a narrowing of the birth canal that makes it difficult to inserted the IUD, so the patient was consulted to have the IUD inserted via operative hysteroscopy. The patient also received progynova therapy 3×1 tab PO and vitamin E 2×500 mg.

## Discussion

Patient came with complaints of not menstruating for 14 months, even though previously she had normal menstrual cycles of 28 days, with a duration of 5 days. This condition is known as secondary amenorrhoea. Secondary amenorrhoea itself is defined as a condition where menstruation does not occur for at least three months after menarche or in women who previously experienced normal menstrual cycles. The prevalence of secondary amenorrhoea is around 2–5%. Secondary amenorrhoea can be caused by various things, one of which is uterine disorder. One of the causes of uterine disorders is the occurrence of intrauterine adhesions (IUA) that is called Asherman’s syndrome. Apart from being marked by menstrual cycle disturbances and evidence of intrauterine adhesions or synechiae, other symptoms that can arise are abdominal pain and cramps which may or may not occur. In this case, the patient only complained about the cessation of menstrual cycles for more than a year. There were no other accompanying complaints in the patient. This can rule out some of the differential diagnoses for secondary amenorrhoea^9–12^.

Proof of intrauterine adhesions is assessed through hysterosalpingography (HSG), sonohysterography (SHG), Saline Infused Sonohysterography, three-dimensional ultrasonography, hysteroscopy, or MRI. The patient underwent a transvaginal ultrasound examination and the results showed the impressions of normal ovarian, hypoplastic uterus, and suspicion of endometrial adhesions. Suspicion of adhesions was then evaluated further through hysteroscopy, which is the gold standard for IUA investigations. The results of the diagnostic hysteroscopy examination showed a severe degree of Asherman’s syndrome. Based on the American Fertility Society (AFS) 1988, a severe Asherman’s syndrome has a score of>9. This assessment consists of clinical symptoms of the menstrual cycle to assess the capacity of the endometrium that still allows the regeneration of post-adhesiolysis, takes into account the post-treatment prognosis, and helps during pre- treatment of adhesion counselling. The total of patient’s AFS was greater than 9, consisting of an assessment of the severity degree, involvement of the uterine cavity in which reached greater than 2/3, heavy adhesions, and cessation of the menstrual cycle (amenorrhoea). The European Society of Hysteroscopy (ESH) 1989, classified the hysteroscopy results of such patients into the grade IV group indicated by the presence of extensive strong adhesions to the uterine wall^13,14^.

The patient was said to have uterine hypoplasia with a length of 6.57 cm, a height of 1.99 cm, a width of 2.28 cm, and a volume of 15.59 cm^3^. According to Michael (2013), the normal size of an adult uterus is on a length range of 7–9 cm, a width of 4.5–6 cm, and a depth of 2.5–3.5 cm^15^. The average normal uterine volume at the age of 16–55 years is around 34–35 cm^3^ ^16^. The hypoplasia is the impact of uterine ischaemia due to b-Lynch compression resulting in thinning of the uterine wall and decreasing the functional capacity. The occurrence of uterine ischaemia is reported to appear as early as 24 h after the procedure. No visible endometrial margin on ultrasound examination with uterine infarction was also demonstrated in some cases. Secondary infertility and oligohypomenorrhea have also been reported previously^17^.

The B-lynch suturing method is a conservative surgical technique for treating uterine atony in PPH. The B-Lynch method has proven successful in stopping bleeding and is recommended in all countries. The success rate is 86.4% in preventing hysterectomy. The anterior and posterior uterine walls will be united through vertical sutures so that the uterus will experience compression. Uterine compression will reduce the volume of the uterine cavity to maintain homoeostasis during bleeding. Although the application of B-Lynch has long been recommended, since it was first introduced by Christopher in 1997 until now, this method is not free from postoperative complications^5,18^.

B-lynch sutures that penetrate the myometrium can cause uterine tension and ischaemia. Suture erosion of the uterine wall is reported as a sequence of myometrial ischaemia which will progress to tissue necrosis. Excessive B-lynch sutures will also affect myometrial and endometrial vascularization. Impaired blood supply leads to hypoperfusion and the exacerbation of ischaemia^17^.

A study conducted by Akoury and Sherman reported that early complications occurred one year after B-lynch which was characterized by the formation of synechiae in the uterus. The finding of uterine synechiae increases by the time after B-lynch. The development of synechiae is suspected as a result of progressive myometrial necrosis caused by compression of the sutures. Synechiae cause the adhesions by blocking uterine blood flow. Adhesions occur in the internal uterine cavity and/or cervical canal. This will cause disruption of the development and function of the uterus resulting in amenorrhoea. In addition, uterine necrosis can also occur due to suturing which is too tight so that it interferes with the uterine blood supply. The impaired perfusion has an impact on cell ischaemia and infarction if not reperfused and ends in cell death^13,18^.

Although the B-Lynch procedure rarely causes complications, this case is relevant with Nalini’s explanation that the complications regarding the combination of B-Lynch suture and vessel ligation or UAE, should be tightening enough the suture in the B-lynch procedure so that the uterine cavity remains completely obliterated, but too tight sutures in the B-Lynch procedure probably can cause the ischaemic necrosis, Asherman syndrome, and infertility. The type of suture used in the B-Lynch suture also might be associated with the complication. Non-absorbable sutures or delayed absorbable sutures might be associate with the highest degree of tightening, and the resultant extreme degree of compression might give rise to more incidences of ischaemic necrosis of the uterine wall, Asherman syndrome, and infertility. The case that reported by Uzel also showed that the main cause of development of Asherman’s syndrome is endometrial trauma. Chaudhary and colleagues also reported the developed of uterine necrosis after B-lynch procedure with gangrene in myometrium and cervix. Wu and Yeh reported uterine synechiae formation following square sutures which were visible two years after procedure through hysteroscopy. Other cases have also been reported of uterine resection after one B-lynch and two Cho sutures. The use of absorbable suture is thought to be one of the causes of uterine necrosis complication. The study from Kwong and colleagues reported two women with Asherman’s syndrome and one mild uterine synechiae. Both of them with Asherman’s syndrome had Hayman sutures applied during their elective caesarean sections. The difference is the first woman also had uterine artery ligations and later pelvic vessel embolization for six hours after operation due to persistent bleeding. We postulated the severe hemodynamic shock and the combination of three devascularization techniques might have contributed to uterine ischaemia and scarring over the endometrium. The second woman underwent Hayman sutures and uterine artery ligations. We postulated that severe hemodynamic, combination of techniques and degree of compression potentiate to cause endometrial scarring and ischaemic damage due to excessive suture tension on the myometrium^2,13,20,21^.

Vital signs within normal limits indicate no signs of a systemic inflammatory response that can occur in patients with infection, pyometra, or uterine abscess after B-lynch. Wenxue and colleagues reported necrotic ischaemic events and secondary infections in patients 4 days after B-Lynch^16^. Physical examination of general status was within normal limits. This is done to remove other co-morbidities that can cause secondary amenorrhoea, for instance thyroid enlargement, visual acuity (related to pituitary tumours), and skin (cortisol hormone disorders). The most common cause of secondary amenorrhoea is pregnancy. This is because the superficial layers of endometrium, which periodically sheds, is supported by progesterone secreted by the corpus luteum after fertilization^22^. Genital status within normal limits showed no clinical signs of hypo or hypergonadism in the patient^11,23^.

There is no suspicion that leads to hyper or hypogonadism, the patient was not tested for testosterone and prolactin^19^. Examination of hormones in the form of FSH, LH, and estradiol showed results within normal limits. This is supported by the results of transvaginal ultrasound and hysteroscopy which showed a normal image of both ovaries of the patient. Normal FSH, LH, and estradiol accompanied by no bleeding response leads to Asherman’s syndrome supported by a history of uterine instrumentation in the patient. The results of other laboratory tests also showed normal values. Normal haematology supports no signs of secondary infection. Infection that occurs can exacerbate adhesions, thus it required prompt diagnosis and treatment^12^.

Based on the results of the anamnesis, physical examination, and other supporting examinations, the patient was diagnosed with severe secondary amenorrhoea et causa Asherman’s syndrome. The patient is then planned to undergo operative hysteroscopy. Operative hysteroscopy is a therapeutic option to treat intrauterine adhesions. This action is indicated in Asherman’s syndrome with menstrual disorders and infertility. Severe adhesion in the patient will complicate and endanger hysteroscopy due to the loss of clear anatomical boundaries, so there is a risk of uterine perforation. Therefore, monitoring during surgery is needed to reduce this risk^24,25,26^.

This case shows that B-lynch still can causes a serious complication although the procedure is recommended to treat PPH. It means that we have to be aware during procedure to minimalize or prevent the complications. Other option that can be considered is the use of compression sutures alone. Fotopoulou and Dudenhausen explained that the combination of compression sutures and additional vessel ligation was more often associated with complications, probably because of the development of uterine ischaemia and inflammation. We can consider to do unilateral ligation to maintain blood supply and prevent total devascularization that can cause ischaemia and necrosis. B-Lynch and Shah explained that unilateral ligation of cervix/vagina, ovarian, and illiaca can stop heavy bleeding without devascularization other organs. The other method to treat uterine necrosis and synechia is by laparoscopic removal and a new removable UCS. In order to prevent endometrial scarring, Kwong and colleagues suggested the application of appropriate tension on uterine walls by suture material which is firm, monofilament and quickly absorbed is of paramount importance. Recently, there were reports of novel compression sutures which were removed in early postoperative period. If they cause severe complications, hysterectomy is required to prevent septicaemia, embolism, and other complications that may endanger the patient’s life^27,28^.

## Conclusion

Our case shows that the B-Lynch procedure, which is the method recommended in worldwide for treating postpartum haemorrhage due to its high success rate, can actually cause complications of Asherman’s syndrome and cause secondary amenorrhoea in this case. The limitation of this study is we did not undergo testosterone and prolactin laboratory test to ensure no leads to hyper or hypogonadism. In addition, we did not know how long the bleeding that the patient experienced at the first hospital was. This is because the duration of PPH is one of the factors that contributes to the outcome of the B-Lynch procedure.

## Ethical approval

Ethical approval for this study (No. KET-896/UN2.F1/ETIK/PPM.00.02/2023) was provided by the Ethical Committee of Faculty Medicine Indonesia University on 03 July 2023.

## Consent

Written informed consent was obtained from the patient for publication and any accompanying images. A copy of the written consent is available for review by the Editor-in-Chief of this journal on request.

## Source of funding

The authors fully financed the funding for this study and does not receive any form of sponsorship or grant.

## Author contribution

A.K.H. conceptualized the idea and gathered the data, P.N. writing the manuscript and managed the resources, A.F.A. analyzed and contributed to writing the manuscript, B.W. extracted and interpreted the results.

## Conflicts of interest disclosure

The authors declare that they have no conflict of interest.

## Guarantor

All authors are guarantor for the study.

## Data availability statement

All data generated or analysed during this study are included in the article and will publicly available.

## Methods

The work has been reported in line with the SCARE criteria.
